# Cross-Correlations and Joint Gaussianity in Multivariate Level Crossing Models

**DOI:** 10.1186/2190-8567-4-22

**Published:** 2014-04-17

**Authors:** Elena Di Bernardino, José León, Tatjana Tchumatchenko

**Affiliations:** 1 Laboratoire Cédric EA4629, Conservatoire National des Arts et Métiers, 292 rue Saint-Martin, Paris Cedex 03, France; 2 Centro de Probabilidades y Estadística, Escuela de Matemáticas, Facultad de Ciencias, Universidad Central de Venezuela, Av. Los Ilustres, Los Chaguaramos AP: 47197, Caracas, Venezuela; 3 Max Planck Institute for Brain Research, Max-von-Laue-Str 4, 60438, Frankfurt am Main, Germany

## Abstract

A variety of phenomena in physical and biological sciences can be mathematically understood by considering the statistical properties of level crossings of random Gaussian processes. Notably, a growing number of these phenomena demand a consideration of correlated level crossings emerging from multiple correlated processes. While many theoretical results have been obtained in the last decades for individual Gaussian level-crossing processes, few results are available for multivariate, jointly correlated threshold crossings. Here, we address bivariate upward crossing processes and derive the corresponding bivariate Central Limit Theorem as well as provide closed-form expressions for their joint level-crossing correlations.

## 1 Introduction

Various phenomena in the biological or physical sciences are amenable to the description by level crossings of random Gaussian processes [1,2]. Examples of these phenomena are spike coordination of neurons in the brain [3], insurance risk assessment [4] and stress levels generated by ocean waves [5]. Therefore a number of mathematical studies in recent decades have focused on the statistical properties of level crossings arising from stationary Gaussian processes [2]. However, largely this literature addresses the properties of one level-crossing process and rarely deals with the coordinated level crossings of multivariate Gaussian processes. A prominent application where correlated level crossings are of particular importance is neuroscience. Recent work has shown that the spikes of a cortical neuron can be approximated by a Gaussian level-crossing process [3,6]. The assumption of Gaussianity is prompted by the experimental observation that cortical neurons are on average connected to ∼10000 neurons and therefore receive a barrage of inputs that together lead to a near-Gaussian fluctuation at the cell body of any given cortical neuron [7]. The spikes of two neurons are then modeled as upward level-crossing times of two cross-correlated fluctuating Gaussian potentials. 

In this article we aim to address two features of level crossings of multiple correlated Gaussian processes. First, we want to clarify whether level-crossing counts derived from multiple correlated processes are jointly Gaussian. Second, we want to understand how many more coincident level crossings in a given time instance are expected if the underlying Gaussian random processes are correlated. Let us provide an intuitive reason for these questions. Starting with the first question, we recognize that if level-crossing counts of two neurons were jointly Gaussian, then a simple measure of dependence is the covariance or the Pearson correlation coefficient. Measuring a vanishing correlation coefficient or vanishing covariance between two neuronal spike counts would in this case imply true statistical independence, because only in the case of multivariate Gaussian distribution is it permissible to conclude independence from vanishing count correlations. This implication is not permissible if the marginal distributions are not Gaussian or are Gaussian but the joint distribution is not a multivariate Gaussian distribution. While marginal Gaussianity has been shown for level-crossing counts in [2] for large bin sizes, joint Gaussianity is still an open question. It might seem natural to imply joint Gaussianity from marginal Gaussianity for multivariate level-crossing processes, however, numerous counter examples exist to prove this intuition wrong, see Sect. 5 in [8]. Here, we use a modified Breuer–Major Theorem to prove joint Gaussianity and show that any linear combination of level-crossing counts of the two processes is also Gaussian. 

The second question we address in this article deals with the conditional probabilities of two level-crossing processes. We are interested in how the level crossings of one Gaussian process can be used to predict the level-crossing probability of the partner process in a specific time interval relative to the observed level crossing in one process. In neuroscience, coordinated neuronal firing drives changes in synaptic connectivity and calculating the spike count dependencies across neurons is therefore a topic of current research efforts (e.g. Chap. 8 in [9]). The available mathematical results for conditional upward crossings in Gaussian processes currently comprise mostly variance and moments for one level-crossing process (see Chaps. 3–5 in [2]) as well as the low and high correlation limit in pairs of processes [3,10]. As yet, a comprehensive closed-form solution covering the complete level-crossing cross-correlation function is currently lacking. Here, we use a regression approach to derive, for all correlation strengths, the conditional level-crossing correlation functions in two continuous Gaussian processes. We hypothesize that the level-crossing correlations we provide in this article could also be valuable in other fields outside of neuroscience for example in risk assessment calculations to predict the risk of joint default for insurance purposes. 

The article is structured as follows. In Sect. 2 we define the mathematical model setting and introduce the concept of level crossings and specifically the upward crossings. In Sect. 3 we use a regression approach to obtain a general closed-form solution for cross-correlations of level crossings in two correlated Gaussian processes. In Sect. 4 we prove the joint Gaussianity (Central Limit Theorem) for the correlated joint upward crossings for two correlated Gaussian processes. In the section on materials and methods (Sect. 6) we provide detailed derivations of the reported results. We assume throughout this article that both level-crossing processes arise from crossings of the same threshold level by two Gaussian processes with different variances. This is permissible because the number of level crossings, the Rice rate [11], depends only on the variance-to-threshold ratio, but not on these quantities individually. We therefore work with a pair of level-crossing processes where each process has a unique voltage variance and therefore the rate of crossings in the two neurons being considered are, unless stated otherwise, not the same. Let us note that this assumption is prompted by the observation that in a living brain typically no two neurons are identical in all their properties and differ at least in their firing rate. 

## 2 Mathematical Definitions of Multivariate Level Crossings

**Fig. 1:**
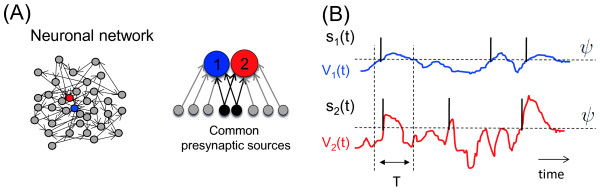
*Cross-correlations in the Gaussian variables lead to correlations of coincident level crossings*. **a** Spike correlations can arise from common input in a neuronal network. **b** We consider coincident level crossings arising from two Gaussian processes that share a common latent source. Whenever the voltage crosses a threshold *ψ* from below a spike is emitted. Spikes are indicated by *vertical solid lines*. *The vertical dotted lines* indicate the width of a time bin *T* used to compute spike counts $U_{\left[0,T\right]}^{V_{i}}(\psi )$, i=1,2

Here we address the statistics of coincident level crossings arising from two Gaussian processes that share a common latent source. This situation is illustrated in Fig. 1(a). We choose to illustrate the situation using a neuroscience perspective. Neurons in a mammalian brain receive synaptic inputs, both excitatory and inhibitory, from thousands of other neurons. Particularly in the visual cortex, the excitatory and inhibitory inputs largely cancel each other and lead to a net fluctuating residual current at the cell body of each neuron. These residual fluctuations drive the spikes of individual neurons. These voltage fluctuations arise from largely independent inputs so they are well approximated by a random Gaussian process with temporal correlations determined by the temporal structure of synaptic currents [7]. 

### 2.1 Definitions of Multivariate Voltage Distributions

We begin by defining the random, temporally correlated Gaussian zero mean process $V_{j}(t)$ which represents the voltage of a neuron *j*

(1) $$V_{j}(t)=\int_{-\infty }^{\infty }e^{it\lambda }f_{V}^{1/2}(\lambda )\left(\sqrt{1-r}dW_{j}(\lambda )+\sqrt{r}dW_{c}(\lambda )\right),$$

where $f_{V}$ is a combination of filters $f_{V}(\lambda )=\gamma (\lambda )g(\lambda )$, where *γ* represents the membrane filter and *g* the synaptic filter. Both of these filters can be chosen freely, but their combination should guarantee a continuously differentiable voltage trajectory. $W_{j}$ with j={1,2} and $W_{c}$ are complex random measures with independent increments, such that for all Borelian sets A∈B(R) we have $E{\left|W(A)\right|}^{2}=m(A)$, the Lebesgue measure of *A*. By $W_{c}$ we denote the common noise component. Moreover if A∩B=∅ then W(A) and W(B) are independent Gaussian random variables. The correlation strength *r*, r=[0,1), denotes the presynaptic overlap of neurons 1 and 2 generated in a neuronal network, and it is illustrated in Fig. 1. If r=0 the voltages $V_{1}$ and $V_{2}$ are independent if r=1 the voltages $V_{1}$ and $V_{2}$ are identical. The auto- and cross-correlation functions between $V_{i}$ and $V_{j}$ are, respectively

(2) $$C_{V_{j}}(\tau )=\langle V_{j}(0)V_{j}(\tau )\rangle =\sigma_{V_{j}}^{2}c(\tau ),$$

(3) $$C_{V_{jk}}(\tau )=\langle V_{j}(0)V_{k}(\tau )\rangle =r\sigma_{V_{j}}\sigma_{V_{k}}c(\tau )\;\text{for }j,k\in \{1,2\},$$

where $c(\tau )=\int_{-\infty }^{\infty }e^{i\tau \lambda }f_{V}(\lambda )d\lambda$, and *τ* is the considered delay. The vector $\left(V_{1}(0),V_{1}^{\prime }(0),V_{2}(\tau ),V_{2}^{\prime }(\tau )\right)$ comprising the voltages and their derivatives is Gaussian and has the covariance matrix

(4) $$\Sigma (\tau )=(\begin{array}{cccc} \sigma_{V_{1}}^{2} & 0 & \Sigma_{13}(\tau ) & \Sigma_{14}(\tau )\\ 0 & \sigma_{V_{1}^{\prime }}^{2} & -\Sigma_{14}(\tau ) & \Sigma_{24}(\tau )\\ \Sigma_{13}(\tau ) & -\Sigma_{14}(\tau ) & \sigma_{V_{2}}^{2} & 0\\ \Sigma_{14}(\tau ) & \Sigma_{24}(\tau ) & 0 & \sigma_{V_{2}^{\prime }}^{2} \end{array}),$$

where the variances are $\sigma_{V_{j}}^{2}=C_{V_{j}}(0)$, $\sigma_{V_{j}^{\prime }}^{2}=-C_{V_{j}}^{\prime \prime }(0)$ and covariance functions are given by

$$\begin{array}{rl} \Sigma_{13}(\tau ) & =\langle V_{1}(0)V_{2}(\tau )\rangle =r\sigma_{V_{1}}\sigma_{V_{2}}c(\tau ),\\ \Sigma_{14}(\tau ) & =\langle V_{1}(0)V_{2}^{\prime }(\tau )\rangle =r\sigma_{V_{1}}\sigma_{V_{2}}c^{\prime }(\tau ),\\ \Sigma_{24}(\tau ) & =\langle V_{1}^{\prime }(0)V_{2}^{\prime }(\tau )\rangle =-r\sigma_{V_{1}}\sigma_{V_{2}}c^{\prime \prime }(\tau ). \end{array}$$

We use the correlation time $\tau_{s}$ to quantify the width of the correlation function c(τ):

(5) $$\tau_{s}=\sqrt{c(\tau )/\left|c^{\prime \prime }(\tau )\right|}.$$

If the filters γ(λ) and g(λ) are classic low-pass filters, then the correlated voltage processes of Eq. (1) can be written in a differential form for each neuron *j*:

(6) $$\tau_{M}V_{j}^{\prime }(t)=-V_{j}(t)+I_{j}(t),$$

where $I_{j}(t)$ is the residual Gaussian current fluctuation with variance *σ*, $\tau_{M}$ the membrane time constant of the neuron, e.g. [12-15]. The synaptic drive $I_{j}(t)$ can be separated into two parts: a common and an individual noise component

(7) $$I_{j}(t)=\int_{-\infty }^{\infty }e^{it\lambda }\sqrt{C_{\xi }(\lambda )}\left(\sqrt{1-r}dW_{i}(\lambda )+\sqrt{r}dW_{c}(\lambda )\right),$$

where $C_{\xi }(\lambda )$ is the synaptic noise spectral density. Using Eq. (6) we obtain the following spectral representation for the stationary solutions:

$$V_{j}(t)=\sigma_{i}\int_{-\infty }^{\infty }e^{it\lambda }\frac{\sqrt{C_{\xi }(\lambda )}}{\left(1+i\tau_{M}\lambda \right)}\left(\sqrt{1-r}dW_{i}(\lambda )+\sqrt{r}dW_{c}(\lambda )\right).$$

In this form the spectral density of each $V_{j}$ is given by $f_{V}(\lambda )=C_{\xi }(\lambda )/(1+\tau_{M}^{2}\lambda^{2})$. Analogously to Eq. (3), we obtain

(8) $$E\left[V_{1}(0)V_{2}(\tau )\right]=r\sigma_{1}\sigma_{2}\int_{-\infty }^{\infty }e^{i\tau \lambda }\frac{C_{\xi }(\lambda )}{\left(1+\tau_{M}^{2}\lambda^{2}\right)}d\lambda =r\sigma_{1}\sigma_{2}c(\tau ).$$

### 2.2 Upward Crossing Definitions

Neurons communicate using brief pulses, the so called *spikes*, which are emitted whenever a voltage threshold is crossed [9]. The integrate-and-fire-type neuron models that are frequently used in computational neuroscience [12-15] generate a spike in neuron *j* any time a voltage $V_{j}(t)$ crosses a fixed threshold *ψ* and subsequently reset the voltage to a reset potential. Recently, it has been shown that in many physiologically relevant cases the leaky integrate-and-fire model can be equivalent to a level-crossing model without reset, where spikes are modeled as positive threshold crossings and are not followed by a reset [3,6,16]. Here, we therefore identify the spikes of a neuron *j* with the positive level crossings of its voltage $V_{j}(t)$ and quantify the cross-correlation between level crossings in neurons 1 and 2 by the following level functional:

(9) $$\begin{array}{rcl} U_{Q(\varepsilon )}(\psi ) & = & \lim_{\delta \to 0}\frac{1}{{\left(2\delta \right)}^{2}}\int_{Q(\varepsilon )}1_{\left\{|V_{1}(s_{1})-\psi |<\delta \right\}}V_{1}^{\prime }(s_{1})1_{\left\{V_{1}^{\prime }(s)\geq 0\right\}}\\ & & \times 1_{\left\{|V_{2}(s_{1}+s_{2})-\psi |<\delta \right\}}V_{2}^{\prime }(s_{1}+s_{2})1_{\left\{V_{2}^{\prime }(s_{1}+s_{2})\geq 0\right\}}ds_{1}ds_{2}, \end{array}$$

where $Q(\varepsilon )=I_{1}\times I_{2}$ is a bounded and finite rectangle in $R^{2}$, *ψ* denotes the voltage threshold in both neurons (see Fig. 1). Here *δ* is introduced to quantify the infinitesimal interval around the threshold *ψ* where a spike takes place. We choose the same threshold for both neurons and two different variances ($\sigma_{V_{1}}\neq \sigma_{V_{2}}$) and keep all other parameters the same. $\sigma_{V_{1}}\neq \sigma_{V_{2}}$ represents the biological situation in which two neurons of the same neuronal type could have differences in the strength of their synaptic input and threshold-to-variance ratio but are exposed to the same temporal background statistics. We will consider the following random field $Z:R^{2}\times \Omega \to R^{2}$, defined as $\left(s_{1},s_{2})\to Z(s_{1},s_{2})=(V_{1}(s_{1}),V_{2}(s_{1}+s_{2})\right)$. *Ω* denotes the probability space; here *Ω* is the Gaussian probability space. The field *Z* is Gaussian and $Z(s_{1},s_{2})$ and $Z(0,s_{2}-s_{1})$ are equal in distribution. We denoted by $p_{s_{2}-s_{1}}(\cdot ,\cdot )$ the bivariate Gaussian density of vector $\left(V_{1}(s_{1}),V_{2}(s_{2})\right)$. If Q(ε)=[t,t+ε]×[τ,τ+ε] and the prerequisites of Theorem 6.2 in [2] are fulfilled we can write

(10) $$\begin{array}{rcl} & & E\left[U_{Q(\varepsilon )}(\psi )\right]\\ & & \;=E[\#\{(s_{1},s_{2})\in Q(\varepsilon ):V_{1}(s_{1})=\psi ,V_{2}(s_{1}+s_{2})=\psi ,\\ & & \;\;1_{V_{1}^{\prime }(s_{1})\geq 0},1_{V_{2}^{\prime }(s_{1}+s_{2})\geq 0}\}] \end{array}$$

(11) $$\begin{array}{rcl} & & \;=\int_{Q(\varepsilon )}E\left[\left|\det Z^{\prime }(s_{1},s_{2})\right|,1_{V_{1}^{\prime }(s_{1})\geq 0},1_{V_{2}^{\prime }(s_{1}+s_{2})\geq 0}|Z(s_{1},s_{2})=(\psi ,\psi )\right]\\ & & \;\;\times p_{s_{2}-s_{1}}(\psi ,\psi )ds_{1}ds_{2} \end{array}$$

(12) $$\begin{array}{rcl} & & \;=\varepsilon \int_{\tau }^{\tau +\varepsilon }E\left[\left|\det Z^{\prime }(0,s_{2})\right|1_{V_{1}^{\prime }(0)\geq 0},1_{V_{2}^{\prime }(s_{2})\geq 0}|Z(0,s_{2})=(\psi ,\psi )\right]\\ & & \;\;\times p_{s_{2}}(\psi ,\psi )ds_{2} \end{array}$$

(13) $$\begin{array}{rcl} & & \;=\varepsilon \int_{\tau }^{\tau +\varepsilon }E\left[\left|V_{1}^{\prime }(0)\right|\left|V_{2}^{\prime }(s_{2})\right|1_{V_{1}^{\prime }(0)\geq 0},1_{V_{2}^{\prime }(s_{2})\geq 0}|V_{1}(0)=\psi ,V_{2}(s_{2})=\psi \right]\\ & & \;\;\times p_{s_{2}}(\psi ,\psi )ds_{2}, \end{array}$$

where the expectation value is denoted by , and $\det(Z(s_{1},s_{2}))$ is the determinant of the correlation matrix for the vector field $Z(s_{1},s_{2})$. Now, we are left to prove the conditions of Theorem 6.2 in [2]. First, we find that conditions (i) and (ii) of Theorem 6.2 are satisfied by definition. Condition (iii) holds because $p_{s_{2}}(\psi ,\psi )$ is not degenerate. If we let $I_{1}$ and $I_{2}$ be two finite and bounded intervals in ℝ, condition (iv) is satisfied because

$$\begin{array}{rcl} & & P\left\{\exists (s_{1},s_{2})\in I_{1}\times I_{2}:Z(s_{1},s_{2})=(\psi ,\psi ),\det Z(s_{1},s_{2})=0\right\}\\ & & \;\leq P\left\{s_{1}\in I_{1}:V_{1}(s_{1})=\psi ,V_{1}^{\prime }(s_{1})=0\right\}+P\left\{s_{2}\in I_{2}:V_{2}(s_{2})=\psi ,V_{2}^{\prime }(s_{2})=0\right\}. \end{array}$$

Here ℙ denotes the probability measure. We can define the correlation of two spike trains as

(14) $$\begin{array}{rcl} \langle s_{1}(t)s_{2}(t+\tau )\rangle & := & \lim_{\epsilon \to 0}\frac{E[U_{Q(\varepsilon )}(\psi )]}{\epsilon^{2}}\\ & = & E\left[V_{1}^{\prime }(0)1_{\left\{V_{1}^{\prime }(0)\geq 0\right\}}V_{2}^{\prime }(\tau )1_{\left\{V_{2}^{\prime }(\tau )\geq 0\right\}}|V_{1}(0)=\psi ,V_{2}(\tau )=\psi \right]\\ & & \times p_{\tau }(\psi ,\psi ) \end{array}$$

(15) $$=\int_{0}^{\infty }\int_{0}^{\infty }{\dot{v}}_{1}{\dot{v}}_{2}p_{\tau }(\psi ,{\dot{v}}_{1},\psi ,{\dot{v}}_{2})d{\dot{v}}_{1}d{\dot{v}}_{2},$$

where $p_{\tau }(\psi ,{\dot{v}}_{1},\psi ,{\dot{v}}_{2})$ is the joint Gaussian density of the vector $\left(V_{1}(0),V_{1}^{\prime }(0),V_{2}(\tau ),V_{2}^{\prime }(\tau )\right)$. The conditional firing rate $\nu_{\mathrm{cond}}(\tau )$ then is

(16) $$\nu_{\mathrm{cond}}(\tau )=\langle s_{1}(t)s_{2}(t+\tau )\rangle /\sqrt{\nu_{1}\nu_{2}},$$

where $\nu_{j}=\frac{\sigma_{V_{j}^{\prime }}}{2\pi \sigma_{V_{j}}}\exp(-\frac{\psi^{2}}{2\sigma_{V_{j}}^{2}})$ is the firing rate of a neuron *j*, for j=1,2. In the next sections we provide closed-form expressions for $\langle s_{1}(t)s_{2}(t+\tau )\rangle$ and $\nu_{\mathrm{cond}}(\tau )$.

## 3 Cross-Correlations of Two Upward Level Crossings

Here, we address $\langle s_{1}(t)s_{2}(t+\tau )\rangle$ and provide a closed-form solution that is valid for any cross-correlation strength *r* between two level-crossing processes and any time delay *τ*.

**Proposition 3.1***Following the steps outlined in the methods section*, *Sect*. 6.1, *we can apply a regression model and Mehler’s Formula* (*see Lemma *10.7 *in*[2]) *to prove that*

(17) $$\langle s_{1}(t)s_{2}(t+\tau )\rangle =C_{\left(a,b\right)}(\tau )p_{\tau }(\psi ,\psi ),$$

*where*$p_{\tau }(\cdot ,\cdot )$*is a joint Gaussian distribution for voltages*$V_{j}$*as defined in Sect*. 2.2 *and*$C_{\left(a,b\right)}(\tau )$*is the series given by*

(18) $$\begin{array}{rcl} C_{\left(a,b\right)}(\tau ) & = & \left[b\bar{\Phi }(b)-\phi (b)\right]\left[a\bar{\Phi }(a)-\phi (a)\right]\sigma_{\epsilon_{1}}(\tau )\sigma_{\epsilon_{2}}(\tau )+\bar{\Phi }(a)\bar{\Phi }(b){\mathrm{Cov}}_{\left(\epsilon_{1},\epsilon_{2}\right)}(\tau )\\ & & +\sum_{n\geq 2}\frac{\phi (a)\phi (b)H_{n-2}(a)H_{n-2}(b){\mathrm{Cov}}_{\left(\epsilon_{1},\epsilon_{2}\right)}{\left(\tau \right)}^{n}}{n!{\left(\sigma_{\epsilon_{1}}(\tau )\sigma_{\epsilon_{2}}(\tau )\right)}^{n-1}}, \end{array}$$

where

(19) $$\sigma_{\epsilon_{1}}(\tau )={\left(\sigma_{V_{1}^{\prime }}^{2}-\left(\alpha_{1}^{2}\sigma_{V_{1}}^{2}+2\alpha_{1}\alpha_{2}\Sigma_{13}(\tau )+\alpha_{2}^{2}\sigma_{V_{2}}^{2}\right)\right)}^{1/2},$$

(20) $$\sigma_{\epsilon_{2}}(\tau )={\left(\sigma_{V_{2}^{\prime }}^{2}-\left(\beta_{1}^{2}\sigma_{V_{1}}^{2}+2\beta_{1}\beta_{2}\Sigma_{13}(\tau )+\beta_{2}^{2}\sigma_{V_{2}}^{2}\right)\right)}^{1/2},$$

(21) $${\mathrm{Cov}}_{\left(\epsilon_{1},\epsilon_{2}\right)}(\tau )=\Sigma_{24}(\tau )-\left(\beta_{1}\alpha_{1}\sigma_{V_{1}}^{2}+(\alpha_{1}\beta_{2}+\alpha_{2}\beta_{1})\Sigma_{13}(\tau )+\alpha_{2}\beta_{2}\sigma_{V_{2}}^{2}\right),$$

and

$$a=\frac{-(\psi (\alpha_{1}+\alpha_{2}))}{\sigma_{\epsilon_{1}}(\tau )},\;\;b=\frac{-(\psi (\beta_{1}+\beta_{2}))}{\sigma_{\epsilon_{2}}(\tau )},$$

with

$$\begin{array}{rcl} \alpha_{1} & = & \frac{\Sigma_{14}(\tau )\Sigma_{13}(\tau )}{\sigma_{V_{1}}^{2}\sigma_{V_{2}}^{2}-\Sigma_{13}{\left(\tau \right)}^{2}},\;\;\alpha_{2}=\frac{-\Sigma_{14}(\tau )\sigma_{V_{1}}^{2}}{\sigma_{V_{1}}^{2}\sigma_{V_{2}}^{2}-\Sigma_{13}{\left(\tau \right)}^{2}},\\ \beta_{1} & = & \frac{\sigma_{V_{2}}^{2}\Sigma_{14}(\tau )}{\sigma_{V_{1}}^{2}\sigma_{V_{2}}^{2}-\Sigma_{13}{\left(\tau \right)}^{2}},\;\;\beta_{2}=\frac{-\Sigma_{14}(\tau )\Sigma_{13}(\tau )}{\sigma_{V_{1}}^{2}\sigma_{V_{2}}^{2}-\Sigma_{13}{\left(\tau \right)}^{2}}. \end{array}$$

*ϕandΦare the standard Gaussian density and distribution*, *respectively*, *and*$\bar{\Phi }=1-\Phi$. $H_{n}(z)={\left(-1\right)}^{n}\frac{d^{n}}{dz^{n}}(e^{-z^{2}/2})e^{z^{2}/2}$*are the Hermite polynomials*. *Note that the first two terms in Eq*. (18) *correspond to truncation orders*n=0*and*n=1, *respectively*.

**Fig. 2:**
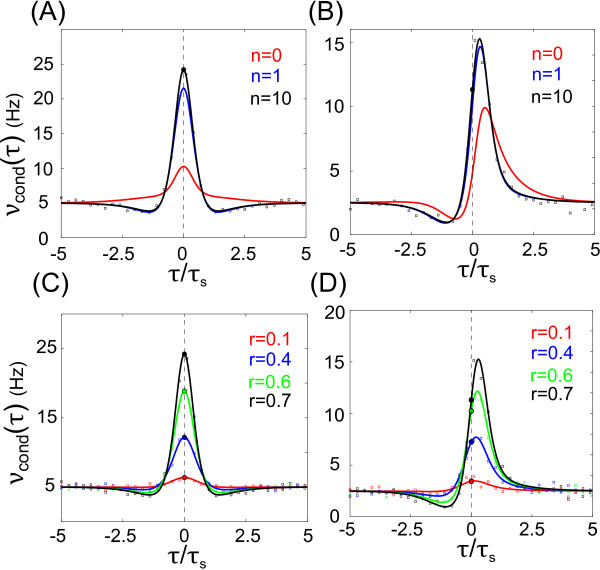
*Convergence of pairwise level crossing correlations*. **a** Spike correlation function $\nu_{\mathrm{cond}}(\tau )$ vs. time lag *τ* for different truncation orders *n* in Eq. (17); r=0.7, $\sigma_{V_{1}}=\sigma_{V_{2}}=10\text{ mV}$, $\tau_{s}=\text{20 ms}$, ν=5 Hz, ψ=9.64 mV. **b**$\nu_{\mathrm{cond}}(\tau )$ vs. *τ* for a pair of rate heterogeneous neurons with r=0.7, $\sigma_{V_{1}}=10\text{ mV}$, $\sigma_{V_{2}}=5\text{ mV}$, $\tau_{s}=20\text{ ms}$, $\nu_{1}=5\text{ Hz}$, $\nu_{2}=1.24\text{ Hz}$, ψ=9.64 mV. **c**$\nu_{\mathrm{cond}}(\tau )$ vs. time lag for varying correlation strengths *r* in a pair of neurons with $\sigma_{V_{1}}=\sigma_{V_{2}}=10\text{ mV}$, $\tau_{s}=20\text{ ms}$, ν=5 Hz, ψ=9.64. **d**$\nu_{\mathrm{cond}}(\tau )$ vs. time lag for varying correlation strengths *r*, in a pair of rate heterogeneous neurons with $\sigma_{V_{1}}=10\text{ mV}$, $\sigma_{V_{2}}=5\text{ mV}$, $\tau_{s}=20\text{ ms}$, $\nu_{1}=5\text{ Hz}$, $\nu_{2}=1.24\text{ Hz}$, ψ=9.64. The truncation order of $\nu_{\mathrm{cond}}(\tau )$-series in Eq. (17) in **c**–**d** is n=10. In **a**–**d***the filled circles* at τ=0 indicate the predicted $\nu_{\mathrm{cond}}(0)$ (as in Eq. (23)) and *colored squares* denote the corresponding numerical simulations obtained with N=2000 independent realizations of 20 s length

To aid the explicit evaluation of $C_{\left(a,b\right)}(\tau )$ in Eq. (18) we provide code for a computer algebra system.^a^ Figure 2(a), (b) demonstrate $\nu_{\mathrm{cond}}(\tau )$ obtained using Eq. (17) for progressively large truncation orders *n*. Notably, we find close correspondence between the first truncation order n=1 and the large *n* limit (n=10). Figure 2(c), (d) show $\nu_{\mathrm{cond}}(\tau )$ vs. *τ* as in Eq. (17) for varying correlation strength *r*. For simplicity, we chose $c(\tau )=\cosh{\left(\tau /\tau_{s}\right)}^{-1}$ and r∈[0,1). We note that $\nu_{\mathrm{cond}}(\tau )$ for two identical neurons ($\sigma_{V_{1}}=\sigma_{V_{2}}$) is a symmetric function while for a pair of neurons with different rates ($\sigma_{V_{1}}\neq \sigma_{V_{2}}$), $\nu_{\mathrm{cond}}(\tau )$ is asymmetric. 

Let us now briefly discuss the result we obtained in Eq. (17) within the context of previous level-crossing literature. One of the closely related results is the variance of level crossings and maxima provided in Proposition 4.4 in [2]. However, this result is derived for one level-crossing process, while we addressed a pair of level-crossing processes. For multiple cross-correlated processes orthant probabilities that describe expressions of the form $P(V_{1}(t)>\psi_{1},V_{2}(t)>\psi_{2})$ have been obtained, e.g. Lemma 4.3 on p. 78 in [2]. However, specific results for the cross-correlations of upward crossings are sparse. For two correlated upward level-crossing processes previous studies have addressed the limiting cases of weak (r≈0) or strong (r≈1, r<1) cross-correlations [3,10]. However, to address upcrossing correlations in the intermediate regimes where neither r≈0 nor r≈1 no analytical methods are available. Therefore, it was previously necessary to numerically evaluate the associated Gaussian probability densities in Eq. (15). The direct numerical evaluation of multidimensional Gaussian integrals can be computationally and algorithmically demanding, requires adaptive grid procedures and its accuracy can be hard to evaluate [17]. For the specific case of τ=0 we show in the materials and methods section on ‘Zero time lag correlations’, Sect. 6.2, that a direct evaluation of the Gaussian double integral is possible via a variable substitution. The key to this variable substitution method was a manageable unity correlation matrix. For any other finite τ>0 and a given finite correlation strength 0≪r<1 we could not identify a transformation that leads to a tractable integral and we therefore derived the series expansion in Eq. (17). This solution is explicit such that each series term of order *n* can be evaluated and studied separately. Furthermore, Eq. (17) consists of analytical functions with a well-studied parameter dependence. This makes it possible to predict the influence of a specific parameter, such as time scale $\tau_{s}$, firing rate $\nu_{i}$ or correlation strength *r* on the upward level-cross correlations. As an example, we evaluate Eq. (18) for an identical pair of neurons up to the third order in *r* via a Taylor expansion. We obtain

(22) $$\begin{array}{rcl} \nu_{\mathrm{cond}}(\tau ) & = & \nu +r\nu \left(c(\tau )\psi^{2}/\sigma_{V}^{2}-\pi \tau_{s}^{2}c^{\prime \prime }(\tau )/2\right)\\ & & +\frac{\nu r^{2}}{2}[c{\left(\tau \right)}^{2}{\left(\frac{\psi^{2}}{\sigma_{V}^{2}}-1\right)}^{2}+\tau_{s}^{2}c^{\prime \prime }(\tau )\left(c^{\prime \prime }(\tau )\tau_{s}^{2}-\pi c(\tau )\frac{\psi^{2}}{\sigma_{V}^{2}}\right)\\ & & +\tau_{s}^{2}c^{\prime }{\left(\tau \right)}^{2}\left(\frac{\psi^{2}}{\sigma_{V}^{2}}(2-\pi )-2\right)]+\nu r^{3}[\frac{c{\left(\tau \right)}^{3}}{3!}{\left(\frac{\psi^{3}}{\sigma_{V}^{3}}-3\frac{\psi }{\sigma_{V}}\right)}^{2}\\ & & +c(\tau )c^{\prime \prime }(\tau )\tau_{s}^{2}\left(c^{\prime \prime }(\tau )\tau_{s}^{2}\frac{\psi^{2}}{2}-\frac{\pi }{4}c(\tau ){\left(\frac{\psi^{2}}{\sigma_{V}^{2}}-1\right)}^{2}\right)\\ & & -\tau_{s}^{2}c(\tau )c^{\prime }{\left(\tau \right)}^{2}\left(\frac{\pi }{2}\left(\frac{\psi^{4}}{\sigma_{V}^{4}}+1\right)+\frac{\psi^{2}}{\sigma_{V}^{2}}\right)]. \end{array}$$

We recognize that the linear and quadratic expressions are equivalent to the first and second order *r*-expansion reported in [3,10]. The cubic term has not been reported before, to the best of our knowledge. This demonstrates consistency with previous results and illustrates that expansions of any order can be obtained via Eq. (18). In this context, it is desirable to have an exact reference point for deciding how many *n*- orders are necessary for Eq. (17) to be sufficiently accurate. Such a reference point can be the zero lag value which we calculate exactly. Deviation of Eq. (17) for a specific *n* from this reference point can serve as an accuracy benchmark. Following the calculations in the methods Sect. 6.2, we derive

(23) $$\begin{array}{rcl} \nu_{\mathrm{cond}}(0) & = & \frac{1+(2r\arctan(\sqrt{\left(1+r)/(1-r\right)}))/\sqrt{1-r^{2}}}{4\pi^{2}\sqrt{\nu_{1}\nu_{2}}\tau_{s}^{2}}\\ & & \times \exp\left(\frac{-\psi^{2}}{4\sigma_{V_{1}}^{2}\sigma_{V_{2}}^{2}}\left[\frac{{\left(\sigma_{V_{1}}+\sigma_{V_{2}}\right)}^{2}}{1+r}+\frac{{\left(\sigma_{V_{2}}-\sigma_{V_{1}}\right)}^{2}}{1-r}\right]\right). \end{array}$$

Figure 2(a), (b) demonstrates $\nu_{\mathrm{cond}}(\tau )$ obtained using Eq. (17) for different truncation orders *n* alongside the zero lag correlation $\nu_{\mathrm{cond}}(0)$. Figure 2(c), (d) demonstrates $\nu_{\mathrm{cond}}(\tau )$ obtained using Eq. (17) as a function of the correlation strength *r* alongside the zero lag correlation $\nu_{\mathrm{cond}}(0)$. As previously, we chose $c(\tau )=\cosh{\left(\tau /\tau_{s}\right)}^{-1}$ and r∈[0,1). We note that for two identical neurons ($\sigma_{V_{1}}=\sigma_{V_{2}}$) $\nu_{\mathrm{cond}}(\tau )$ is a symmetric function. Yet, for a pair of neurons with different rates ($\sigma_{V_{1}}\neq \sigma_{V_{2}}$) the spike correlation function $\nu_{\mathrm{cond}}(\tau )$ is asymmetric, indicating that the lower rate neuron spikes on average after the higher rate neuron.

### 3.1 Relation to the Leaky Integrate-and-Fire Model

Here, we address the relation between spike statistics and spike correlations in the level-crossing setting in our article and previous results in the leaky integrate-and-fire framework [12-15]. In a current-based leaky integrate-and-fire model driven by fluctuating noise the voltage of a neuron $V_{j}(t)$ is given by

(24) $$\tau_{M}V_{j}^{\prime }(t)=-V_{j}(t)+I_{j}(t),$$

(25) $$\tau_{I}I_{j}^{\prime }(t)=-I_{j}(t)+\sigma \xi (t),$$

where $I_{j}(t)$ is the input current of a neuron, ξ(t) a white noise, unit variance drive. The voltage power spectrum for this model is a combination of low-pass filters $f_{V}(\lambda )∼{\left[(1+\tau_{M}^{2}\lambda^{2})(1+\tau_{I}^{2}\lambda^{2})\right]}^{-1}$ and its correlation function can be determined according to Eqs. (8). If the voltage $V_{j}$ reaches the threshold *ϕ* the neuron *j* emits a spike and the voltage is subsequently reset to a reset value $V_{r}$. The integrate-and-fire model differs only in one important detail from the level-crossing approach—the presence of a reset after a spike. A recent article by Laurent Badel systematically compared the validity of upward level-crossing approximation for the firing rate, spike correlations and frequency response of a leaky integrate-and-fire neuron [16]. This study reached the conclusion that the upward level-crossing approach accurately represents the leaky integrate-and-fire model if two conditions are fulfilled: (1) the firing rate is much lower than the typical relaxation time of the voltage, (2) the synaptic filtering time constants remain of the same order of magnitude as the membrane time constant ($\tau_{I}/\tau_{M}\approx 1$). Numerically, the validity of the approximation remained highly accurate even for synaptic time constants $0.4≲\tau_{I}/\tau_{M}≲2.6$.

**Fig. 3:**
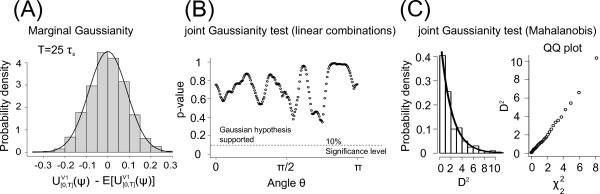
*Univariate and multivariate count Gaussianity*. **a** Univariate Gaussianity of counts $U_{\left[0,T\right]}^{V_{1}}(\psi )-E[U_{\left[0,T\right]}^{V_{1}}(\psi )]$ for large bin size $T=25\tau_{s}$. *A solid black line* represents the corresponding zero mean Gaussian fit. **b** Shapiro test *p*-value of the projections $A=X_{i}^{T}\cdot (\cos(\theta ),\sin(\theta ))$, for all θ∈[0,2π). **c** Probability density of $D^{2}$ (*left*). QQ-plot between the theoretically predicted $\chi_{2}^{2}$-quantiles and the empirical quantiles of $D_{i}^{2}$ in Eq. (35). Figures **b** and **c** are both validations of the multivariate Gaussianity of the bivariate vector **X** in Eq. (34). In all *panels*ψ=0.3, $\tau_{s}=1\text{ ms}$, $\sigma_{V_{i}}=1$

**Fig. 4:**
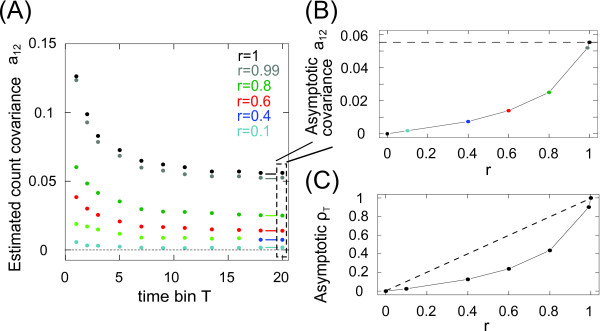
*Finite asymptotic limit of covariances*$a_{ij}$*and correlation coefficient*$\rho_{T}$. **a** Count covariance $a_{12}=\sum_{q=1}^{\infty }\sigma_{X_{1},X_{2}}^{2}(q)$ vs. time bin *T* for varying correlation strengths *r* in the case of two identical neurons. The covariances converge for increasing time bin *T* towards the value predicted in Theorem 4.1 (indicated by *small, thick lines*). Here $\tau_{s}=1$, and ψ=0.3. **b** Limit value of the covariance $a_{12}$ vs. *r* for $T=20\tau_{s}$ (from **a**) vs. *r*. The case of r=1 (*black*) corresponds to the variance $a_{11}\approx 5.5\cdot 10^{-3}$, indicated by *the dashed horizontal line*. **c** Correlation coefficient $\rho_{T}$ vs. *r* for large time bins ($T=20\tau_{s}$). *The dashed line* indicates the equality line

A number of spike correlation results have been derived in the leaky integrate-and-fire model for the limit of weak correlations [18-20]. They include the observation that the spike correlation coefficient increases with firing rate [18,19]. The equivalent firing rate dependent increase in spike correlations and correlation coefficients for low correlation strengths has been reported for the level-crossing model, see [3] and Fig. 3(A) (right) and Fig. 2(A) (top) in [10]. Furthermore, leaky integrate-and-fire model exhibits a sublinear dependence of correlation coefficients on input strength *r*[18,21], which we see confirmed in Fig. 4.

## 4 Joint Gaussianity of Upcrossing Counts

Spike count cross correlations and correlation coefficient measurements in pairs of neurons are ubiquitous in neuroscience and are often used to measure the strength of interdependencies in a pair of neurons, e.g. in cortical neurons [18,19,22], in model neurons [23] and in theoretical and experimental studies of net correlations emerging in recurrent networks [23-28]. Spike counts and their cross correlations in neuroscience are often computed for a variety of bin sizes varying from T=0.1–1 ms[22] to T=2 s[29]. Here, we are interested in the question when spike count correlations of two neurons computed in a bin size *T* are jointly Gaussian such that their cross correlations are unbiased measures of statistical dependence or independence.

In this section we will prove that the spike counts of two neurons, approximated by up crossings of a Gaussian process, approach a joint multivariate Gaussian distribution for large bin sizes *T*. We start by considering the marginal distributions of upcrossing counts. From the one-dimensional Central Limit Theorem proven in [2] we know that $\frac{1}{\sqrt{T}}(U_{\left[0,T\right]}^{V_{j}}(\psi )-E[U_{\left[0,T\right]}^{V_{j}}(\psi )])$ converges for T→∞ to a one-dimensional centered normal variable with finite variance. We provide a direct illustration of this classical univariate result in Fig. 3(a). Now, it is tempting to conclude that because the distribution of counts in each neuron is Gaussian, the joint distribution for the vector $\left(U_{\left[0,T\right]}^{V_{1}}(\psi ),U_{\left[0,T\right]}^{V_{2}}(\psi )\right)$ is also a multivariate Gaussian distribution. Yet, this conclusion is mathematically forbidden. While a joint Gaussian distribution implies marginal Gaussianity it is general not possible to inverse this relation [8]. The joint Gaussianity of spike counts is a highly desired property. If two counts are jointly Gaussian zero count correlation directly implies statistical independence. If count correlations between neuron 1 and neuron 2 are zero such that $U_{\left[0,T\right]}^{V_{1}}(\psi )$ and $U_{\left[0,T\right]}^{V_{2}}(\psi )$ are uncorrelated, then only if the vector $\left(U_{\left[0,T\right]}^{V_{1}}(\psi ),U_{\left[0,T\right]}^{V_{2}}(\psi )\right)$ is from a multivariate Gaussian distribution is it possible to infer independence of neuron 1 and neuron 2. Let us consider a teaching counter example where vanishing correlation between the variables *X* and *Y* does not imply independence: X∈N(0,1) and $Y=X^{2}$. We obtain Cov(X,Y)=0, but the two random variables are strongly linked. Contrasting examples of where *X* and *Y* variables are both marginally but not jointly Gaussian, have a zero correlation but are not independent can be found in Sect. 5 in [8]. To benefit from joint Gaussianity and be able to infer true statistical independence from vanishing correlations, we prove here the joint Gaussianity of upward level crossings/spike counts for large *T*. In the following we derive the Central Limit Theorem for the spike counts of two neurons, using two steps. First, we use the one-dimensional Central Limit Theorem proven in [2]. Second, we apply a version of the Breuer–Major Theorem adapted to our upward crossing setting which we present in Sect. 6.4. 

**Theorem 4.1***Let*$V_{i}(t)$, i∈{1,2}*be two processes satisfying Eq*. (1), *with covariance*$C_{V_{ij}}(\tau )=E[V_{i}(0)V_{j}(\tau )]$*where*i,j=1,2*and a common spiking thresholdψ*. *To take advantage of the available Gaussianity proofs that are typically derived for unit variance processes*, *we rescale the voltages*$V_{i}(t)$*and the thresholdψto obtain processes*$X_{i}(t)$*with unit variance and unit variance of the derivatives*. $X_{i}=\frac{V_{i}(\sqrt{C_{V_{ii}}(0)/|C_{V_{ii}}^{\prime \prime }(0)|}t)}{\sqrt{C_{V_{ii}}(0)}}$*then has the correlation function*$c(\tau )=\frac{C_{V_{ii}}(\sqrt{C_{V_{ii}}(0)/|C_{V_{ii}}^{\prime \prime }(0)|}\tau )}{C_{V_{ii}}(0)}$*and the spiking thresholds are*$\psi_{i}=\psi /\sqrt{C_{V_{ii}}(0)}$. *The number of*$\psi_{i}$-*level upcrossings in a time intervalTfor process*$X_{i}$*is given by*$U_{\left[0,T\right]}^{X_{i}}(\psi_{i})$. *We assume that the following necessary and sufficient conditions hold*. *First*, $E\{{\left(U_{\left[0,T\right]}^{X_{i}}(\psi )\right)}^{2}\}<\infty$. *This holds if and only if*c(τ)*satisfies Geman’s Condition* (*see*, *e*.*g*., *Theorem *3 *in*[30]). *Second*, $\int_{0}^{\infty }|c^{\left(n\right)}(\tau )|d\tau <\infty$*where*n∈{0,1,2}*is the order of derivation*. *Then*

(26) $$\frac{1}{\sqrt{T}}(\begin{array}{c} U_{\left[0,T\right]}^{X_{1}}(\psi_{1})-E[U_{\left[0,T\right]}^{X_{1}}(\psi_{1})]\\ U_{\left[0,T\right]}^{X_{2}}(\psi_{2})-E[U_{\left[0,T\right]}^{X_{2}}(\psi_{2})] \end{array})\overset{d}{\underset{T\to \infty }{\to }}N\left((\begin{array}{c} 0\\ 0 \end{array}),(\begin{array}{cc} a_{11} & a_{12}\\ a_{12} & a_{22} \end{array})\right),$$

*where the count covariances*$a_{ij}$*with*i,j∈{1,2}*are three convergent series*. *Each is then given by*$0<a_{ii}=\sum_{q=1}^{\infty }\sigma_{X_{i}}^{2}(q)$*and*$0<a_{12}=\sum_{q=1}^{\infty }\sigma_{X_{1},X_{2}}(q)$, *both of which are finite*. *The first two terms in these series for*$\psi =\psi_{i}$, $C_{V_{11}}(0)=C_{V_{22}}(0)$*are*

(27) $$\sigma_{X_{1}}^{2}(1)=2\phi {\left(\psi \right)}^{2}\left[\frac{\psi^{2}}{2\pi }\int_{0}^{\infty }c(s)ds-\frac{1}{4}\int_{0}^{\infty }c^{\prime \prime }(s)ds\right],$$

(28) $$\begin{array}{rcl} \sigma_{X_{1}}^{2}(2) & = & 2\phi {\left(\psi \right)}^{2}[\frac{1}{2\pi }{\left(\psi^{2}-1\right)}^{2}\int_{0}^{\infty }c{\left(s\right)}^{2}ds\\ & & +\left(\left(\frac{1}{\pi }-\frac{1}{4}\right)\psi^{2}-\frac{1}{\pi }\right)\int_{0}^{\infty }c^{\prime }{\left(s\right)}^{2}ds \end{array}$$

(29) $$+\frac{1}{2\pi }\int_{0}^{\infty }c^{\prime \prime }{\left(s\right)}^{2}ds-\frac{1}{4}\psi^{2}\int_{0}^{\infty }c(s)c^{\prime \prime }(s)ds],$$

and the first and second order covariances are

(30) $$\sigma_{X_{1},X_{2}}(1)=2\phi {\left(\psi \right)}^{2}r\left[\frac{\psi^{2}}{2\pi }\int_{0}^{\infty }c(s)ds-\frac{1}{4}\int_{0}^{\infty }c^{\prime \prime }(s)ds\right],$$

(31) $$\begin{array}{rcl} \sigma_{X_{1},X_{2}}(2) & = & 2\phi^{2}(\psi )r^{2}[\frac{1}{2\pi }{\left(\psi^{2}-1\right)}^{2}\int_{0}^{\infty }c{\left(s\right)}^{2}ds\\ & & +\left(\left(\frac{1}{\pi }-\frac{1}{4}\right)\psi^{2}-\frac{1}{\pi }\right)\int_{0}^{\infty }c^{\prime }{\left(s\right)}^{2}ds \end{array}$$

(32) $$+\frac{1}{2\pi }\int_{0}^{\infty }c^{\prime \prime }{\left(s\right)}^{2}ds-\frac{1}{4}\psi^{2}\int_{0}^{\infty }c(s)c^{\prime \prime }(s)ds].$$

*We note that only for*r=1*we obtain*$\sigma_{X_{1},X_{2}}(j)=\sigma_{X_{1}}^{2}(j)$. *The asymptotic Pearson correlation coefficient*$\rho_{T}$, *defined by*

(33) $$\lim_{T\to \infty }\rho_{T}=\frac{\mathrm{Cov}(U_{\left[0,T\right]}^{X_{1}}(\psi_{1}),U_{\left[0,T\right]}^{X_{2}}(\psi_{2}))}{\sqrt{\mathrm{Var}(U_{\left[0,T\right]}^{X_{1}}(\psi_{1}))}\sqrt{\mathrm{Var}(U_{\left[0,T\right]}^{X_{2}}(\psi_{2}))}}=\frac{a_{12}}{\sqrt{a_{11}a_{22}}},$$

*will also converge to the respective ratio of the asymptotic covariances and variances*.

### 4.1 Numerical Confirmation of Joint Gaussianity and Limit Covariances $a_{ij}$

In the last section we showed that spike counts of two neurons in large bins approach a bivariate Gaussian distribution with finite variances. Here, we illustrate this theoretical result in simulations of level-crossing processes. We choose two methods based on linear combinations and the Mahalanobis distance to numerically confirm joint Gaussianity. First, we numerically confirm the joint Gaussianity by showing that all linear combinations of two simulated spike counts for a large bin are Gaussian. We consider a vector

(34) $$\text{X}:=\left(\frac{1}{\sqrt{T}}\left(U_{\left[0,T\right]}^{X_{1}}(\psi )-E\left[U_{\left[0,T\right]}^{X_{1}}(\psi )\right]\right),\frac{1}{\sqrt{T}}\left(U_{\left[0,T\right]}^{X_{2}}(\psi )-E\left[U_{\left[0,T\right]}^{X_{2}}(\psi )\right]\right)\right)$$

where $U_{\left[0,T\right]}^{X_{1}}(\psi )$, $U_{\left[0,T\right]}^{X_{2}}(\psi )$ and $E[U_{\left[0,T\right]}^{X_{1}}(\psi )]$, $E[U_{\left[0,T\right]}^{X_{2}}(\psi )]$ are the spike counts and their average in neuron 1 and 2, respectively. **X** consists of *N* × two-dimensional samples. *N* denotes the number of sample realizations and i∈[1,N] the consecutive sample number. We project each two-dimensional element ${\text{X}}_{i}$ in all directions (cos(θ),sin(θ)) by calculating the scalar product $A=X_{i}^{T}\cdot (\cos(\theta ),\sin(\theta ))$, with θ∈[0,2π). Subsequently, we apply a Shapiro test on *A* to verify Gaussianity for all univariate projections. The *p*-value of this Shapiro test conveys the certainty with which the Gaussian hypothesis cannot be rejected. As a second numerical test of joint Gaussianity we use the Mahalanobis distance. This test is based on the fact that if $\text{X}∼N_{d}(\mu ,\Sigma )$, where *d* is the dimensionality, *μ* is the mean and *Σ* the standard deviation, then the Mahalanobis distance $D^{2}$ with entries $D_{i}^{2}$

(35) $$D_{i}^{2}=\left\{{\left(X_{i}-\mu \right)}^{\prime }\Sigma^{-1}(X_{i}-\mu ),i=1,\ldots ,n\right\},$$

is distributed according to a $\chi_{d}^{2}$-distribution with *d* degrees of freedom (see, e.g., Sect. 3.1.4 and Eq. (3.16) in [4]). By numerically estimating the count sample average *μ* and *Σ* we calculate in our case $D_{i}^{2}$ and compare it with a $\chi_{2}^{2}$-distribution, using the QQ-plot method (see Fig. 3(c)).

Figure 3 demonstrates the results of the joint Gaussianity tests for a bin size $T=25\tau_{s}$, where $\tau_{s}=1\text{ ms}$. Figure 3(a) shows the empirical univariate distribution of spike counts in one level-crossing process derived from N=10000 independent count realizations. Figure 3(b) demonstrates that in N=10000 independent count realizations of **X***p*-values for all *θ* are above the 10 % significance level. Figure 3(c) (left) illustrates that the Mahalanobis distance $D^{2}$ of a two-dimensional spike count variable **X** are well approximated by the $\chi_{2}^{2}$-distribution (solid line). Figure 3(c) (right) demonstrates in a QQ-plot of the empirically measured $D^{2}$-quantiles and the theoretical $\chi_{2}^{2}$-quantiles that they are linearly related. This is an indication that both distributions are equal.

Figure 4 addresses the numerical confirmation of the constant asymptotic covariances $a_{ij}$ introduced in Theorem 4.1 in Eq. (26). We choose ψ=0.3, $\tau_{s}=1$, for N=5000 independent count realizations. To numerically compute the covariances $a_{ij}$ from spike count simulations we used the covariance-matrix estimator proposed by [31]. Figure 4(a) demonstrates the convergence of covariance $a_{12}$ to a finite value that is predicted by Theorem 4.1. The asymptotic large *T* limit is denoted by a brief colored horizontal line. Figure 4(b) demonstrates the dependence of this asymptotic limit on the correlation strength *r*. As expected, we find that the asymptotic estimated covariance $a_{12}$ is close to zero for r=0 and it is close to the variance for r=1. Figure 4(c) demonstrates that the interplay between covariances $a_{12}$ and variances $a_{11}$ and $a_{22}$ leads to a sublinear dependence of the asymptotic correlation coefficient $\rho_{T}=a_{12}/(\sqrt{a_{11}a_{22}})$ (Eq. (33)) in the large time bin limit, $T=25\tau_{s}$. 

## 5 Conclusions

Level-crossing phenomena occur in a variety of physical and biological sciences. In many of these situations coordination between level crossings of multiple cross-correlated Gaussian processes is of interest. Here, we focused on neuroscience and modeled the spikes of two cross-correlated neurons by two cross-correlated level-crossing processes. While crossings and extrema of one level-crossing process have been the focus of mathematical research, results describing the coordination of multiple level-crossing processes are sparse and typically available only in specific and limited cases. Limits where level-crossing cross-correlations have been previously calculated are the weak and strong input correlation limit [3]. Here, we studied the case of two cross-correlated upward crossing processes and derived closed-form expressions for their joint level-crossing coordination as well their joint count Gaussianity. Importantly, the results we present in this article are consistent with previously reported limits but we now extended and generalized them. The two main results of our article are (1) closed-form explicit solution of the level-crossing cross-correlations and (2) the joint Gaussian limit of level-crossing counts. Our first result provides an explicit solution to $\nu_{\mathrm{cond}}(\tau )=\langle s_{1}(t)s_{2}(t+\tau )\rangle /\sqrt{\nu_{1}\nu_{2}}$ that is valid for all correlation strengths and which comprises previously obtained limits, see discussion in Sect. 3. The rate of level crossings by a one-dimensional Gaussian process is given by the prominent Rice’s equation derived by Rice in the 1950s [11]. The solution we obtained for the level-crossing cross-correlation $\nu_{\mathrm{cond}}(\tau )$ extends the Rice rate to the joint rate of two correlated processes. Our second result proves the joint Gaussianity of level crossings for large bin sizes. The joint Gaussianity of spike counts is a highly desired property because if and only if two level-crossing counts are jointly Gaussian can zero count cross-correlation imply statistical independence. Notably, marginal Gaussianity of spike counts in each neuron combined with zero count cross-correlation is not sufficient to imply independence. Contrasting examples of where *X* and *Y* variables are both marginally but not jointly Gaussian, have a zero cross-correlation but are not independent can be found in Sect. 5 in [8]. Count covariance and measures derived from it, such as the Pearson correlation coefficient, are computationally inexpensive and widely used as measures of statistical interdependencies [8]. Therefore, it is highly desirable to investigate the joint Gaussianity of level counts and thereby delimit the parameter space and mathematical conditions ensuring that independence can be implied from zero correlation coefficient. Notably, the joint Gaussianity of spike counts in bins of size *T* where *T* is much larger than the intrinsic time constant $\tau_{s}$ ($T\gg \tau_{s}$) also implies that models of multi-neuronal dynamics only need to consider the mean and variance of spike counts because all higher cumulants are zero.

## 6 Materials and Methods

### 6.1 Proof of Proposition 3.1

For simplicity of notation we adopt the following convention: $\left(X_{1},Y_{1},X_{2},Y_{2}\right)$ denotes the vector $\left(V_{1}(0),V_{1}^{\prime }(0),V_{2}(\tau ),V_{2}^{\prime }(\tau )\right)$. In order to calculate $\langle s_{1}(t)s_{2}(t+\tau )\rangle$, defined in (14), we use the following regression model:

$$\left\{ \begin{array}{c} Y_{1}=\alpha_{1}X_{1}+\alpha_{2}X_{2}+\epsilon_{1},\\ Y_{2}=\beta_{1}X_{1}+\beta_{2}X_{2}+\epsilon_{2}, \end{array}\right.$$

where $\left(\epsilon_{1},\epsilon_{2}\right)$ and $\left(X_{1},X_{2}\right)$ are independent. We use the covariance matrix in (4) and obtain

$$\begin{array}{rcl} \alpha_{1} & = & \frac{\Sigma_{14}(\tau )\Sigma_{13}(\tau )}{\sigma_{X_{1}}^{2}\sigma_{X_{2}}^{2}-\Sigma_{13}{\left(\tau \right)}^{2}},\;\;\alpha_{2}=\frac{-\Sigma_{14}(\tau )\sigma_{V_{1}}^{2}}{\sigma_{X_{1}}^{2}\sigma_{X_{2}}^{2}-\Sigma_{13}{\left(\tau \right)}^{2}},\\ \beta_{1} & = & \frac{\sigma_{X_{2}}^{2}\Sigma_{14}(\tau )}{\sigma_{X_{1}}^{2}\sigma_{X_{2}}^{2}-\Sigma_{13}{\left(\tau \right)}^{2}},\;\;\beta_{2}=\frac{-\Sigma_{13}(\tau )\Sigma_{14}(\tau )}{\sigma_{X_{1}}^{2}\sigma_{X_{2}}^{2}-\Sigma_{13}{\left(\tau \right)}^{2}}. \end{array}$$

The conditional distribution $L(Y_{1},Y_{2}|X_{1}=\psi ,X_{2}=\psi )$ is a bivariate Gaussian distribution

(36) $$N\left(\begin{array}{c} \psi (\alpha_{1}+\alpha_{2})\\ \psi (\beta_{1}+\beta_{2}) \end{array},(\begin{array}{cc} \sigma_{\epsilon_{1}}{\left(\tau \right)}^{2} & {\mathrm{Cov}}_{\left(\epsilon_{1},\epsilon_{2}\right)}(\tau )\\ {\mathrm{Cov}}_{\left(\epsilon_{1},\epsilon_{2}\right)}(\tau ) & \sigma_{\epsilon_{2}}{\left(\tau \right)}^{2} \end{array})\right).$$

Using the regression system above, we write the conditional expectation

(37) $$\begin{array}{rl} & E[Y_{1}1_{\left\{Y_{1}\in [0,\infty )\right\}}Y_{2}1_{\left\{Y_{2}\in [0,\infty )\right\}}|X_{1}=\psi ,X_{2}=\psi ]\\ & \;=E[1_{\left\{\epsilon_{1}\in [-\psi (\alpha_{1}+\alpha_{2}),\infty ]\right\}}1_{\left\{\epsilon_{2}\in [-\psi (\beta_{1}+\beta_{2}),\infty ]\right\}}\\ & \;\;\cdot \left(\psi^{2}(\alpha_{1}+\alpha_{2})(\beta_{1}+\beta_{2})+\epsilon_{1}\psi (\beta_{1}+\beta_{2})+\epsilon_{2}\psi (\alpha_{1}+\alpha_{2})+\epsilon_{1}\epsilon_{2}\right)]\\ & \;=\sigma_{\epsilon_{1}}(\tau )\sigma_{\epsilon_{2}}(\tau )(abE[1_{\left\{Z_{1}\in [a,\infty )\right\}}1_{\left\{Z_{2}\in [b,\infty )\right\}}]-bE[Z_{1}1_{\left\{Z_{1}\in [a,\infty )\right\}}1_{\left\{Z_{2}\in [b,\infty )\right\}}]\\ & \;\;-aE[Z_{2}1_{\left\{Z_{1}\in [a,\infty )\right\}}1_{\left\{Z_{2}\in [b,\infty )\right\}}]+E[Z_{1}1_{\left\{Z_{1}\in [a,\infty )\right\}}Z_{2}1_{\left\{Z_{2}\in [b,\infty )\right\}}]), \end{array}$$

where $Z_{1}=\frac{\epsilon_{1}}{\sigma_{\epsilon_{1}}(\tau )}$, $Z_{2}=\frac{\epsilon_{2}}{\sigma_{\epsilon_{2}}(\tau )}$, $a=\frac{-(\psi (\alpha_{1}+\alpha_{2}))}{\sigma_{\epsilon_{1}}(\tau )}$, $b=\frac{-(\psi (\beta_{1}+\beta_{2}))}{\sigma_{\epsilon_{2}}(\tau )}$. Now, we calculate the four different expectations in Eq. (37) using Mehler’s Formula (Lemma 10.7 in [2]). First, we write

(38) $$E[Z_{1}1_{\left\{Z_{1}\in [a,\infty )\right\}}Z_{2}1_{\left\{Z_{2}\in [b,\infty )\right\}}]=\sum_{n=0}^{\infty }c_{n}(a)c_{n}(b)n!{\left(\frac{{\mathrm{Cov}}_{\left(\epsilon_{1},\epsilon_{2}\right)}(\tau )}{\sigma_{\epsilon_{1}}(\tau )\sigma_{\epsilon_{2}}(\tau )}\right)}^{n},$$

where $c_{n}(a)$ and $c_{n}(b)$ are the Hermite coefficients associated with the expectation $E[Z_{1}1_{\left\{Z_{1}\in [a,\infty )\right\}}Z_{2}1_{\left\{Z_{2}\in [b,\infty )\right\}}]$. Then these Hermite coefficients are given by

$$c_{n}(a)=\frac{{\left(-1\right)}^{n}}{n!\sqrt{2\pi }}\int_{a}^{\infty }z\frac{d^{n}}{dz^{n}}\left(e^{-z^{2}/2}\right)dz,\;\;c_{n}(b)=\frac{{\left(-1\right)}^{n}}{n!\sqrt{2\pi }}\int_{b}^{\infty }z\frac{d^{n}}{dz^{n}}\left(e^{-z^{2}/2}\right)dz.$$

In particular, 

– for n=0, $c_{0}(a)=\frac{e^{-a^{2}/2}}{\sqrt{2\pi }}=\phi (a)$, $c_{0}(b)=\phi (b)$,

– for n=1, $c_{1}(a)=a\phi (a)-\Phi (a)+1$, $c_{1}(b)=b\phi (b)-\Phi (b)+1$,

– for n≥2, $c_{n}(a)=\frac{\phi (a)}{n!}(aH_{n-1}(a)+H_{n-2}(a))$, $c_{n}(b)=\frac{\phi (b)}{n!}(bH_{n-1}(b)+H_{n-2}(b))$.

 Analogously we obtain

(39) $$E[1_{\left\{Z_{1}\in [a,\infty )\right\}}1_{\left\{Z_{2}\in [b,\infty )\right\}}]=\sum_{n=0}^{\infty }c_{n}(a)c_{n}(b)n!{\left(\frac{{\mathrm{Cov}}_{\left(\epsilon_{1},\epsilon_{2}\right)}(\tau )}{\sigma_{\epsilon_{1}}(\tau )\sigma_{\epsilon_{2}}(\tau )}\right)}^{n},$$

with

$$c_{n}(a)=\frac{{\left(-1\right)}^{n}}{n!\sqrt{2\pi }}\int_{a}^{\infty }\frac{d^{n}}{dz^{n}}\left(e^{-z^{2}/2}\right)dz,\;\;c_{n}(b)=\frac{{\left(-1\right)}^{n}}{n!\sqrt{2\pi }}\int_{b}^{\infty }\frac{d^{n}}{dz^{n}}\left(e^{-z^{2}/2}\right)dz.$$

In particular, 

– for n=0, $c_{0}(a)=1-\Phi (a)=\bar{\Phi }(a)$, $c_{0}(b)=\bar{\Phi }(b)$,

– for n=1, $c_{1}(a)=\phi (a)$, $c_{1}(b)=\phi (b)$,

– for n≥2, $c_{n}(a)=\frac{\phi (a)}{n!}H_{n-1}(a)$, $c_{n}(b)=\frac{\phi (b)}{n!}H_{n-1}(b)$.

 We also have

(40) $$E[1_{\left\{Z_{1}\in [a,\infty )\right\}}Z_{2}1_{\left\{Z_{2}\in [b,\infty )\right\}}]=\sum_{n=0}^{\infty }c_{n}(a)c_{n}(b)n!{\left(\frac{{\mathrm{Cov}}_{\left(\epsilon_{1},\epsilon_{2}\right)}(\tau )}{\sigma_{\epsilon_{1}}(\tau )\sigma_{\epsilon_{2}}(\tau )}\right)}^{n},$$

with

$$c_{n}(a)=\frac{{\left(-1\right)}^{n}}{n!\sqrt{2\pi }}\int_{a}^{\infty }\frac{d^{n}}{dz^{n}}\left(e^{-z^{2}/2}\right)dz,\;\;c_{n}(b)=\frac{{\left(-1\right)}^{n}}{n!\sqrt{2\pi }}\int_{b}^{\infty }z\frac{d^{n}}{dz^{n}}\left(e^{-z^{2}/2}\right)dz.$$

In particular, 

– for n=0, $c_{0}(a)=\bar{\Phi }(a)$, $c_{0}(b)=\phi (b)$,

– for n=1, $c_{1}(a)=\phi (a)$, $c_{1}(b)=b\phi (b)-\Phi (b)+1$,

– for n≥2, $c_{n}(a)=\frac{\phi (a)}{n!}H_{n-1}(a)$, $c_{n}(b)=\frac{\phi (b)}{n!}(bH_{n-1}(b)+H_{n-2}(b))$.

 Finally,

(41) $$E[Z_{1}1_{\left\{Z_{1}\in [a,\infty )\right\}}1_{\left\{Z_{2}\in [b,\infty )\right\}}]=\sum_{n=0}^{\infty }c_{n}(a)c_{n}(b)n!{\left(\frac{{\mathrm{Cov}}_{\left(\epsilon_{1},\epsilon_{2}\right)}(\tau )}{\sigma_{\epsilon_{1}}(\tau )\sigma_{\epsilon_{2}}(\tau )}\right)}^{n},$$

with

$$c_{n}(a)=\frac{{\left(-1\right)}^{n}}{n!\sqrt{2\pi }}\int_{a}^{\infty }z\frac{d^{n}}{dz^{n}}\left(e^{-z^{2}/2}\right)dz,\;\;c_{n}(b)=\frac{{\left(-1\right)}^{n}}{n!\sqrt{2\pi }}\int_{b}^{\infty }\frac{d^{n}}{dz^{n}}\left(e^{-z^{2}/2}\right)dz.$$

In particular, 

– for n=0, $c_{0}(a)=\phi (a)$, $c_{0}(b)=\bar{\Phi }(b)$,

– for n=1, $c_{1}(a)=a\phi (a)-\Phi (a)+1$, $c_{1}(b)=\phi (b)$,

– for n≥2, $c_{n}(a)=\frac{\phi (a)}{n!}(aH_{n-1}(a)+H_{n-2}(a))$, $c_{n}(b)=\frac{\phi (b)}{n!}H_{n-1}(b)$.

 Then, combining the four expectations (38)–(41), the associated Hermite coefficients, and Eq. (37), we obtain

(42) $$\begin{array}{rl} & E[Y_{1}1_{\left\{Y_{1}\in [0,\infty )\right\}}Y_{2}1_{\left\{Y_{2}\in [0,\infty )\right\}}|X_{1}=\psi ,X_{2}=\psi ]\\ & \;=\left(b\bar{\Phi }(b)-\phi (b)\right)\left(a\bar{\Phi }(a)-\phi (a)\right)\sigma_{\epsilon_{1}}(\tau )\sigma_{\epsilon_{2}}(\tau )\\ & \;\;+\bar{\Phi }(a)\bar{\Phi }(b){\mathrm{Cov}}_{\left(\epsilon_{1},\epsilon_{2}\right)}(\tau )\\ & \;\;+\sum_{n\geq 2}\frac{{\mathrm{Cov}}_{\left(\epsilon_{1},\epsilon_{2}\right)}{\left(\tau \right)}^{n}}{n!{\left(\sigma_{\epsilon_{1}}(\tau )\sigma_{\epsilon_{2}}(\tau )\right)}^{n-1}}\left(\phi (a)\phi (b)H_{n-2}(a)H_{n-2}(b)\right). \end{array}$$

Note that the first two terms in Eq. (42) correspond to orders n=0 and n=1, respectively. Denoting $E[Y_{1}1_{\left\{Y_{1}\in [0,\infty )\right\}}Y_{2}1_{\left\{Y_{2}\in [0,\infty )\right\}}|X_{1}=\psi ,X_{2}=\psi ]=C_{\left(a,b\right)}(\tau )$ we find $\langle s_{1}(t)s_{2}(t+\tau )\rangle =C_{\left(a,b\right)}(\tau )p_{\tau }(\psi ,\psi )$. Here, $C_{\left(a,b\right)}(\tau )$ is a uniformly convergent series.  □

### 6.2 Zero Time Lag Correlations

In this section we derive the zero lag spike correlation using the Gaussian probability integrals in Eq. (15). Following the previously introduced notation the spiking threshold level in a neurons is *ψ* and variance $\sigma_{V_{i}}$ we can write

$$\begin{array}{rcl} \nu_{\mathrm{cond}}(\tau ) & = & \langle s_{1}(t)s_{2}(t)\rangle /(\nu_{1}\nu_{2})\\ & = & \frac{1}{\sqrt{\nu_{1}\nu_{2}}}\int_{0}^{\infty }\int_{0}^{\infty }{\dot{V}}_{1}\cdot {\dot{V}}_{2}(t+\tau )p_{\tau }(\psi ,\psi ,{\dot{V}}_{1},{\dot{V}}_{2})d{\dot{V}}_{1}d{\dot{V}}_{2}(\tau ). \end{array}$$

Now, we substitute the variables:

$$\begin{array}{rcl} \Sigma & = & \frac{\psi (\sigma_{V_{1}}+\sigma_{V_{2}})}{\sqrt{2\sigma_{V_{1}}\sigma_{V_{2}}(r\sigma_{V_{1}}\sigma_{V_{2}}+\sigma_{V_{1}}\sigma_{V_{2}})}},\\ \dot{\Sigma } & = & \frac{\sqrt{\sigma_{{\dot{V}}_{2}}/\sigma_{{\dot{V}}_{1}}}\dot{V}(t)+\sqrt{\sigma_{{\dot{V}}_{1}}/\sigma_{{\dot{V}}_{2}}}\dot{V}(t+\tau )}{\sqrt{2}\sqrt{\sigma_{{\dot{V}}_{1}}\sigma_{{\dot{V}}_{2}}-r\sigma_{V_{1}}\sigma_{V_{2}}c^{\prime \prime }(\tau )}},\\ \Delta & = & \frac{\psi (\sigma_{V_{2}}-\sigma_{V_{1}})}{\sqrt{2\sigma_{V_{1}}\sigma_{V_{2}}(\sigma_{V_{1}}\sigma_{V_{2}}-r\sigma_{V_{1}}\sigma_{V_{2}})}},\\ \dot{\Delta } & = & \frac{\sqrt{\sigma_{{\dot{V}}_{2}}/\sigma_{{\dot{V}}_{1}}}\dot{V}(t)-\sqrt{\sigma_{{\dot{V}}_{1}}/\sigma_{{\dot{V}}_{2}}}\dot{V}(t+\tau )}{\sqrt{2}\sqrt{\sigma_{{\dot{V}}_{1}}\sigma_{{\dot{V}}_{2}}+r\sigma_{V_{1}}\sigma_{V_{2}}c^{\prime \prime }(\tau )}}. \end{array}$$

The correlation matrix $C_{\Sigma ,\dot{\Delta },\dot{\Sigma },\Delta }$ for τ=0 is a four-dimensional identity matrix.

(43) $$\begin{array}{rcl} \nu_{\mathrm{cond}}(0) & = & \frac{\sigma_{{\dot{V}}_{1}}^{2}\sigma_{{\dot{V}}_{2}}^{2}}{\sqrt{\nu_{1}\nu_{2}}}\frac{\sqrt{1-r^{2}}}{8\pi^{2}(1-r^{2})\sigma_{V_{1}}\sigma_{V_{2}}\sigma_{{\dot{V}}_{1}}\sigma_{{\dot{V}}_{2}}}\\ & & \times \int_{-\infty }^{\infty }[\exp\left(-\frac{{\dot{\Delta }}^{2}(1-r)}{2(1+r)}\right)|\dot{\Delta }|\sqrt{\left(1-r^{2}\right)}\\ & & +\sqrt{\frac{\pi }{2}}\left[(1+r)-{\dot{\Delta }}^{2}(1-r)\right]\mathrm{Erfc}\left(|\frac{\dot{\Delta }\sqrt{\left(1-r\right)}}{\sqrt{2}\sqrt{\left(1+r\right)}}|\right)]\\ & & \cdot \exp(-\frac{\psi^{2}{\left(\sigma_{V_{1}}+\sigma_{V_{2}}\right)}^{2}}{4\sigma_{V_{1}}\sigma_{V_{2}}(r\sigma_{V_{1}}\sigma_{V_{2}}+\sigma_{V_{1}}\sigma_{V_{2}})}\\ & & -\frac{\psi^{2}{\left(\sigma_{V_{2}}-\sigma_{V_{1}}\right)}^{2}}{4\sigma_{V_{1}}\sigma_{V_{2}}(\sigma_{V_{1}}\sigma_{V_{2}}-r\sigma_{V_{1}}\sigma_{V_{2}})}-\frac{{\dot{\Delta }}^{2}}{2})d\dot{\Delta }. \end{array}$$

Solving this integral we obtain Eq. (23).

### 6.3 Proof of Theorem 4.1

To prove joint Gaussianity, it is instructive to briefly recapitulate the one-dimensional Central Limit Theorem. We start by writing

(44) $$U_{\left[0,T\right]}^{X_{i}}(\psi_{1})=\sum_{j=0}^{\infty }\sum_{k=0}^{\infty }d_{j}^{\left(i\right)}(\psi_{i})a_{k}\int_{0}^{T}H_{j}\left(X_{i}(s)\right)H_{k}\left(X_{i}^{\prime }(s)\right)ds,$$

with $d_{j}^{\left(i\right)}(\psi_{i})=\frac{1}{j!}\phi (\psi_{i})H_{j}(\psi_{i})$ and $a_{k}=\frac{1}{k!}\int_{0}^{\infty }xH_{k}(x)\phi (x)dx$. Defining the level count deviation for neuron *i* by $S^{i}(T)$ we obtain

(45) $$\begin{array}{rcl} S^{i}(T) & = & \frac{1}{\sqrt{T}}\left(U_{\left[0,T\right]}^{X_{i}}(\psi_{i})-E\left[U_{\left[0,T\right]}^{X_{i}}(\psi )\right]\right)\\ & = & \sum_{q=1}^{\infty }\frac{1}{\sqrt{T}}\int_{0}^{T}\sum_{k+j=q}d_{j}^{\left(i\right)}(\psi_{i})a_{k}H_{j}\left(X_{i}(s)\right)H_{k}\left(X_{i}^{\prime }(s)\right)ds \end{array}$$

(46) $$=\sum_{q=1}^{\infty }\frac{1}{\sqrt{T}}\int_{0}^{T}G_{q}^{i}\left(X_{i}(s),X_{i}^{\prime }(s)\right)ds$$

(47) $$=\sum_{q=1}^{\infty }J_{q}^{i}\left(T,X_{i},X_{i}^{\prime }\right),$$

where $G_{q}^{i}(x_{1},x_{2})=\sum_{k+j=q}d_{j}^{\left(i\right)}(\psi_{i})a_{k}H_{j}(x_{1})H_{k}(x_{2})$. A Gaussian distribution is a stable limit distribution for a sum of independent finite variance variables. Therefore, all that is left to prove is that contributions $q\neq q^{\prime }$ are independent and have finite variance. From Mehler’s Formula we recognize that the contributions for $q\neq q^{\prime }$ are independent. The finite variance follows from the observation that for all *q* the variance of $G_{q}^{i}(X_{i}(s),X_{i}^{\prime }(s))$ is proportional to the expectation of a product of four Hermite polynomials, which has been proven to be finite (Theorem 10.10 in [2]) if the conditions of Theorem 4.1 are satisfied. 

Now, we address the joint Gaussianity. A random vector is jointly Gaussian if and only if any linear combination of its components has a univariate normal distribution (see [4,32,33]). Thus, we need to prove that the sequence $\alpha_{1}S^{1}(T)+\alpha_{2}S^{2}(T)$ is asymptotically Gaussian and satisfies a Central Limit Theorem, for all $\alpha_{1},\alpha_{2}\in R$. We start from Eq. (45) and consider a truncated series for $S_{Q}^{i}(T)=\sum_{q=1}^{Q}J_{q}^{1}(T,X_{1},X_{1}^{\prime })$ consisting of the first *Q* terms and denote by ∥⋅∥ the norm in $L^{2}(\Omega )$. First, we show that the remainder is bounded

(48) $$\begin{array}{rl} & \lim_{T\to \infty }∥\sum_{i=1}^{2}\alpha_{i}S^{i}(T)-\sum_{i=1}^{2}\alpha_{i}S_{Q}^{i}(T)∥\\ & \;\leq |\alpha_{1}|\sqrt{\sum_{q=Q+1}^{\infty }\sigma_{X_{1}}^{2}(q)}+|\alpha_{2}|\sqrt{\sum_{q=Q+1}^{\infty }\sigma_{X_{2}}^{2}(q)}. \end{array}$$

As *Q* grows, this difference diminishes such that if $\alpha_{1}S_{Q}^{1}(T)+\alpha_{2}S_{Q}^{2}(T)$ is Gaussian for large *Q* then this will imply that $\alpha_{1}S^{1}(T)+\alpha_{2}S^{2}(T)$ is Gaussian, too. Now, we only need to show Gaussianity of $\alpha_{1}S_{Q}^{1}(T)+\alpha_{2}S_{Q}^{2}(T)$. Using a modified version of the Breuer–Major Theorem (Sect. 6.4), we know that for each *q*

$$\alpha_{1}J_{q}^{1}\left(T,X_{1},X_{1}^{\prime }\right)+\alpha_{2}J_{q}^{2}\left(T,X_{2},X_{2}^{\prime }\right)\overset{d}{\underset{T\to \infty }{\to }}N\left(0,\sigma_{{\tilde{G}}_{q}}^{2}\right),$$

where $\sigma_{{\tilde{G}}_{q}}^{2}$ is given in Eq. (51). The same theorem implies that $\alpha_{1}J_{q}^{1}(T,X_{1},X_{1}^{\prime })+\alpha_{2}J_{q}^{2}(T,X_{2},X_{2}^{\prime })$ and $\alpha_{1}J_{q^{\prime }}^{1}(T,X_{1},X_{1}^{\prime })+\alpha_{2}J_{q^{\prime }}^{2}(T,X_{2},X_{2}^{\prime })$ are asymptotically independent if $q\neq q^{\prime }$. Thus we obtain for any Q≥1

$$\alpha_{1}S_{Q}^{1}(T)+\alpha_{2}S_{Q}^{2}(T)\overset{d}{\underset{T\to \infty }{\to }}N\left(0,\sum_{q=1}^{Q}\sigma_{{\tilde{G}}_{q}}^{2}\right).$$

To calculate the count covariances in Eq. (26) we use Lemma 10.7 in [2] and obtain

(49) $$\begin{array}{rcl} a_{ij} & = & 2\sum_{q=1}^{\infty }\sum_{k=0}^{q}\sum_{k^{\prime }=0}^{q}d_{q-k}^{\left(1\right)}(\psi_{i})d_{q-k^{\prime }}^{\left(2\right)}(\psi_{j})a_{k}a_{k^{\prime }}\\ & & \times \int_{0}^{\infty }E\left[H_{q-k}\left(X_{i}(0)\right)H_{k}\left(X_{i}^{\prime }(0)\right)H_{q-k^{\prime }}\left(X_{j}(s)\right)H_{k^{\prime }}\left(X_{j}^{\prime }(s)\right)\right]ds. \end{array}$$

Applying the Mehler Formula to a four-dimensional Gaussian random vector (Lemma 10.7 in [2]) we find

(50) $$\begin{array}{rcl} & & E\left[H_{q-k}\left(X_{i}(0)\right)H_{k}\left(X_{i}^{\prime }(0)\right)H_{q^{\prime }-k^{\prime }}\left(X_{j}(s)\right)H_{k^{\prime }}\left(X_{j}^{\prime }(s)\right)\right]\\ & & \;=\{\begin{array}{cc} 0, & \text{if }q\neq q^{\prime },\\ {\left(r^{q}\right)}^{1-\delta_{ij}}\sum_{(d_{1},d_{2},d_{3},d_{4})\in Z}\frac{(q-k)!k!(q-k^{\prime })!k^{\prime }!}{d_{1}!d_{2}!d_{3}!d_{4}!} & \\ \;\cdot c{\left(s\right)}^{d_{1}}c^{\prime }{\left(s\right)}^{d_{2}}{\left(-c^{\prime }(s)\right)}^{d_{3}}{\left(-c^{\prime \prime }(s)\right)}^{d_{4}}, & \text{if }q=q^{\prime }. \end{array} \end{array}$$

Here, $\delta_{ij}$ is the Kronecker delta, *Z* is the set of $d_{i}$’s satisfying: $d_{i}\geq 0$, $d_{1}+d_{2}=q-k$, $d_{3}+d_{4}=k$, $d_{1}+d_{3}=q-k^{\prime }$, and $d_{2}+d_{4}=k^{\prime }$. We thus can express the covariances of the spike counts as

$$\begin{array}{rcl} a_{ij} & = & 2\sum_{q=1}^{\infty }{\left(r^{q}\right)}^{1-\delta_{ij}}\sum_{k=0}^{q}\sum_{k^{\prime }=0}^{q}d_{q-k}^{\left(1\right)}(\psi )d_{q-k^{\prime }}^{\left(2\right)}(\psi )a_{k}a_{k^{\prime }}\\ & & \times \int_{0}^{\infty }\sum_{d_{1},d_{2},d_{3},d_{4}\in Z}\frac{(q-k)!k!(q-k^{\prime })!k^{\prime }!}{d_{1}!d_{2}!d_{3}!d_{4}!}\\ & & \cdot c{\left(s\right)}^{d_{1}}c^{\prime }{\left(s\right)}^{d_{2}}{\left(-c^{\prime }(s)\right)}^{d_{3}}{\left(-c^{\prime \prime }(s)\right)}^{d_{4}}ds. \end{array}$$

This is the result reported in Theorem 4.1.  □

### 6.4 Modified Breuer–Major Theorem

Here, we adapt the Breuer–Major Theorem [34] to show that the bivariate vector $\left(J_{q}^{1}(T,X_{1},X_{1}^{\prime }),J_{q}^{2}(T,X_{2},X_{2}^{\prime })\right)$ is Gaussian.

**Theorem 6.1***We consider two zero mean and unit variance Gaussian processes*$X_{i}(t)$, *which are described by the properties in Sect*. 2 *and a correlation function*$E[X_{i}(0)X_{j}(t)]=c_{ij}(t)$, *where*i,j∈{1,2}. *For all functions*$G_{i}(\cdot ,\cdot )$*that fulfill*$E[G_{i}(X_{i}(0),X_{i}^{\prime }(0))]=0$*and*$E[G_{i}^{2}(X_{i}(0),X_{i}^{\prime }(0))]<\infty$*and two real constants*$\alpha_{i}$*the following integral is Gaussian and we have*

$$\frac{1}{\sqrt{T}}\int_{0}^{T}\left(\alpha_{1}G_{1}\left(X_{1}(t),X_{1}^{\prime }(t)\right)+\alpha_{2}G_{2}\left(X_{2}(t),X_{2}^{\prime }(t)\right)\right)dt\overset{d}{\underset{T\to \infty }{\to }}N\left(0,\sigma_{G}^{2}\right),$$

where

(51) $$\begin{array}{rcl} \sigma_{G}^{2} & = & 2\alpha_{1}^{2}\int_{0}^{\infty }E\left[G_{1}\left(X_{1}(0),X_{1}^{\prime }(0)\right)G_{1}\left(X_{1}(t),X_{1}^{\prime }(t)\right)\right]dt\\ & & +2\alpha_{2}^{2}\int_{0}^{\infty }E\left[G_{2}\left(X_{2}(0),X_{2}^{\prime }(0)\right)G_{2}\left(X_{2}(t),X_{2}^{\prime }(t)\right)\right]dt\\ & & +4\alpha_{1}\alpha_{2}\int_{0}^{\infty }E\left[G_{1}\left(X_{1}(0),X_{1}^{\prime }(0)\right)G_{2}\left(X_{2}(t),X_{2}^{\prime }(t)\right)\right]dt. \end{array}$$

*Proof* We start by considering the Hermite expansion of the functions $G_{i}(\cdot ,\cdot )$ and write

(52) $$G_{i}\left(X_{1}(t),X_{2}^{\prime }(t)\right)=\lim_{Q\to \infty }\sum_{q=1}^{Q}\sum_{k_{1}+k_{2}=q}c_{k_{1}k_{2},G_{i}}H_{k_{1}}\left(X_{1}(t)\right)H_{k_{2}}\left(X_{2}^{\prime }(t)\right)$$

(53) $$=\lim_{Q\to \infty }G_{i}^{Q},$$

where $G_{i}^{Q}$ denotes the sum over *q* in Eq. (52) truncated at *Q*, and the convergence is in $L^{2}(\Omega )$. We write

(54) $$\frac{1}{\sqrt{T}}\int_{0}^{T}\alpha_{1}G_{1}\left(X_{1}(t),X_{1}^{\prime }(t)\right)+\alpha_{2}G_{2}\left(X_{2}(t),X_{2}^{\prime }(t)\right)dt$$

(55) $$\;=\lim_{Q\to \infty }\frac{1}{\sqrt{T}}\int_{0}^{T}\left(\alpha_{1}G_{1}^{Q}\left(X_{1}(t),X_{1}^{\prime }(t)\right)+\alpha_{2}G_{2}^{Q}\left(X_{2}(t),X_{2}^{\prime }(t)\right)\right)dt.$$

Now, to prove the Gaussianity of Eq. (54) it is sufficient to prove the asymptotic Gaussianity of $\left(\alpha_{1}G_{1}^{Q}(X_{1}(t),X_{1}^{\prime }(t))+\alpha_{2}G_{2}^{Q}(X_{2}(t),X_{2}^{\prime }(t))\right)$. This is sufficient because

(56) $$K_{Q}(T)=∥\frac{1}{\sqrt{T}}\int_{0}^{T}\left[\sum_{i=1}^{2}\alpha_{i}\left(G_{i}\left(X_{i}(t),X_{i}^{\prime }(t)\right)-G_{i}^{Q}\left(X_{i}(t),X_{i}^{\prime }(t)\right)\right)\right]dt∥$$

(57) $$\leq \sum_{i=1}^{2}|\alpha_{i}|∥\frac{1}{\sqrt{T}}\int_{0}^{T}\left[\left(G_{i}\left(X_{i}(t),X_{i}^{\prime }(t)\right)-G_{i}^{Q}\left(X_{i}(t),X_{i}^{\prime }(t)\right)\right)\right]dt∥,$$

where ∥⋅∥ is the norm in $L^{2}(\Omega )$. Each of the terms is bounded,

(58) $$\begin{array}{rcl} & & {∥\frac{1}{\sqrt{T}}\int_{0}^{T}\left[G_{i}\left(X_{i}(t),X_{i}^{\prime }(t)\right)-G_{i}^{Q}\left(X_{i}(t),X_{i}^{\prime }(t)\right)\right]dt∥}^{2}\\ & & \;\leq 2\sum_{Q+1}^{\infty }\int_{0}^{a}\left(1-\frac{t}{T}\right)\sum_{k_{1}+k_{2}=q}c_{k_{1}k_{2},G_{i}}^{2}k_{1}!k_{2}! \end{array}$$

(59) $$\;\;+\sum_{Q+1}^{\infty }\int_{a}^{T}\left(1-\frac{t}{T}\right)\psi {\left(t\right)}^{Q}\sum_{k_{1}+k_{2}=q}c_{k_{1}k_{2},G_{i}}^{2}k_{1}!k_{2}!$$

(60) $$\begin{array}{rcl} & & \;\leq a\sum_{Q+1}^{\infty }\sum_{k_{1}+k_{2}=q}c_{k_{1}k_{2},G_{i}}^{2}k_{1}!k_{2}!\\ & & \;\;+\sum_{Q+1}^{\infty }\int_{a}^{T}\left(1-\frac{t}{T}\right)\psi {\left(t\right)}^{Q}\sum_{k_{1}+k_{2}=q}c_{k_{1}k_{2},G_{i}}^{2}k_{1}!k_{2}!, \end{array}$$

where $\psi (t)=\max(|c_{11}(t)|+|c_{12}(t)|,|c_{21}(t)|+|c_{22}(t)|)$ and *a* is a positive real number such that ψ(t)<1 whenever t>a. Since Eq. (60) is a vanishing series, $\lim_{Q\to \infty }\lim_{T\to \infty }K_{Q}(T)=0$. Now, we address the Gaussianity of $\left(\alpha_{1}G_{1}^{Q}(X_{1}(t),X_{1}^{\prime }(t))+\alpha_{2}G_{2}^{Q}(X_{2}(t),X_{2}^{\prime }(t))\right)$. A result of Kratz and León, Lemma 3, p. 653 in [35], implies that

$$L^{i}(T)=\frac{1}{\sqrt{T}}\int_{0}^{T}G_{i}^{Q}\left(X_{i}(t),X_{i}^{\prime }(t)\right)dt$$

can be approached in $L^{2}(\Omega )$ by the sequence of *ϵ*-dependent processes $X_{i}^{\varepsilon }(t)$$L^{i,\varepsilon }(T)=\frac{1}{\sqrt{T}}\int_{0}^{T}G_{i}^{Q}(X_{i}^{\varepsilon }(t),{\left(X_{i}^{\varepsilon }\right)}^{\prime }(t))dt$. The $\frac{1}{\varepsilon }$-dependent processes for i=1,2, are defined as

(61) $$X_{i}^{\varepsilon }(t)=\int_{-\infty }^{\infty }e^{it\lambda }{\left(f_{V}*\beta_{\varepsilon }(\lambda )\right)}^{1/2}\left(\sqrt{1-r}dW_{j}(\lambda )+\sqrt{r}dW_{3}(\lambda )\right),$$

where ∗ denotes a convolution, $\beta_{\varepsilon }$ is $\beta_{\varepsilon }(t)=\frac{1}{\varepsilon }\beta (\frac{t}{\varepsilon })$, *β* being a positive function with $\int_{-\infty }^{\infty }{\left|\lambda \right|}^{j}|\beta (\lambda )d\lambda <\infty$, j=1,2 and such that its Fourier transform has support in [−1,1], where $E[X_{i}^{\varepsilon }(0)X_{i}^{\varepsilon }(\tau )]=c_{jj}(\tau )\hat{\beta }(\varepsilon \tau )$ these processes are $\frac{1}{\varepsilon }$-dependent. Then

(62) $$\begin{array}{rcl} & & ∥\frac{1}{\sqrt{T}}\int_{0}^{T}\sum_{i=1}^{2}\alpha_{i}G_{i}^{Q}\left(X_{i}(t),X_{i}^{\prime }(t)\right)dt-\alpha_{i}L^{i,\epsilon }(T)∥\\ & & \;\leq \sum_{i=1}^{2}|\alpha_{i}|∥L^{i}(T)-L^{i,\epsilon }(T)∥, \end{array}$$

(63) $$\lim_{\epsilon \to 0}\lim_{T\to \infty }\sum_{i=1}^{2}|\alpha_{i}|∥L^{i}(T)-L^{i,\epsilon }(T)∥=0.$$

This follows from the Central Limit Theorem for $\frac{1}{\varepsilon }$-dependent random vectors and concludes the proof. □

## Competing interests

The authors declare that they have no competing interests.

## Authors’ contributions

The main idea of this paper was proposed jointly by EB, JL and TT. All authors contributed equally to the writing of this paper performed all the steps of the proofs in this research. All authors read and approved the final manuscript.
